# Reproductive Factors but Not Hormonal Factors Associated with Thyroid Cancer Risk: A Systematic Review and Meta-Analysis

**DOI:** 10.1155/2015/103515

**Published:** 2015-08-03

**Authors:** Yijuan Cao, Zengyan Wang, Juan Gu, Fangfang Hu, Yujuan Qi, Qianqian Yin, Qingqing Sun, Guotao Li, Bin Quan

**Affiliations:** ^1^Reproductive Center, Central Hospital of Xuzhou, Affiliated Xuzhou Hospital of Southeast University, Xuzhou 221009, China; ^2^Department of Anesthesia, People's Hospital of Zhucheng, Zhucheng 262200, China; ^3^Reproductive Center, The Affiliated Hospital of Weifang Medical University, Weifang 261000, China; ^4^Department of General Surgery, Central Hospital of Xuzhou, Affiliated Xuzhou Hospital of Southeast University, Xuzhou 221009, China

## Abstract

Many studies have investigated the association between hormonal and reproductive factors and thyroid cancer risk but provided contradictory and inconclusive findings. This review was aimed at precisely estimating this association by pooling all available epidemiological studies. 25 independent studies were retrieved after a comprehensive literature search in databases of PubMed and Embase. Overall, common hormonal factors including oral contraceptive and hormone replacement therapy did not alter the risk of thyroid cancer. Older age at menopause was associated with weakly increased risk of thyroid cancer in overall analysis (RR = 1.24, 95% CI 1.00–1.53, *P* = 0.049); however, longer duration of breast feeding was related to moderately reduced risk of thyroid cancer, suggested by pooled analysis in all cohort studies (RR = 0.7, 95% CI 0.51–0.95, *P* = 0.021). The pooled RR in hospital-based case-control studies implicated that parous women were more susceptible to thyroid cancer than nulliparous women (RR = 2.30, 95% CI 1.31–4.04, *P* = 0.004). The present meta-analysis suggests that older age at menopause and parity are risk factors for thyroid cancer, while longer duration of breast feeding plays a protective role against this cancer. Nevertheless, more relevant epidemiological studies are warranted to investigate roles of hormonal and reproductive factors in thyroid carcinogenesis.

## 1. Introduction

Thyroid cancer is the most common type of endocrine malignancy, which accounts for nearly 3% of all malignancies [1]. Despite low mortality rate, rates of local recurrence and distant metastases are high in thyroid cancer patients. The incidence of thyroid cancer has been increasing worldwide for the last five years, while the etiology remains largely unknown. Ionizing radiation is a well documented risk factor for thyroid cancer [2]. However, not all individuals exposed to radiation develop this disease, implicating some other unknown factors involved in thyroid carcinogenesis, such as hormone-related factors.

Gender discrepancy is well known in thyroid malignancies. Thyroid cancer occurs three times more frequently in women than in men, and the incidence decreases among postmenopausal women. It has been well established that female sex hormones, particularly estrogens, can influence the proliferation and invasion of thyroid cancer cells by recognizing corresponding hormonal receptors expressed in those cells, such as estrogen receptor alpha and beta [3–5]. It has been demonstrated that the secretion of thyroid stimulating hormone (TSH) increased during puberty, pregnancy, and oral contraceptive use [6]. Elevated TSH production can promote thyroid growth, while estrogens increase levels of TSH in human body [7]. Therefore, regulation between TSH and estrogens may play a critical role in the development of thyroid disease, thyroid malignancies in particular. Taken together, it can be hypothesized that some hormonal and reproductive factors may confer modifying effects on thyroid carcinogenesis by influencing the signaling of sex hormones and their receptors in thyroid gland. Many epidemiological studies have investigated roles of hormonal and reproductive factors in the development of thyroid cancer, for instance, oral contraceptive use, hormone replacement therapy, menstrual factors, and fertility status [8–32]. Nevertheless, the precise association has not yet been fully elucidated due to conflicting and inconclusive findings in previous studies. We performed this meta-analysis by pooling all currently published studies to obtain a better estimation and provide important insights into the etiology of thyroid cancer.

## 2. Materials and Methods 

### 2.1. Search Strategy

We searched studies on the association between hormonal and reproductive factors and thyroid cancer risk in PubMed and Embase databases from their inception up to September 10, 2014, using the following items: thyroid cancer, or thyroid carcinoma; and oral contraceptive, hormone replacement therapy, reproductive factors, menstrual factors, age at menarche, age at first birth, menopausal status, age at menopause, parity, pregnancy, reproductive history, or breast feeding; and incidence, or risk factor. References of relevant studies were also screened for additional papers. If studies were duplicated, only the most complete study was included.

### 2.2. Inclusion Criteria

The included studies must conform to the following inclusion criteria: (1) studies on the association of hormonal and reproductive factors with thyroid cancer risk; (2) cohort or case-control studies; (3) publications presenting odds ratios (ORs), relative risks (RRs), or hazard ratios (HRs) with 95% confidence intervals (95% CIs). Studies not associated with hormonal and reproductive factors and thyroid cancer risk, case-only, animal research, case reports, and duplicated studies were all excluded.

### 2.3. Data Extraction

Two investigators independently extracted data from each study by use of the following terms: name of first author, year of publication, study design, country of origins, sample size, study period, matching or adjusted factors, and RRs or HRs or ORs with 95% CIs for the estimation of thyroid cancer risk related to hormonal and reproductive factors. Disagreements were solved by discussion.

### 2.4. Statistical Analysis

Roles of hormonal and reproductive factors in thyroid cancer risk were assessed by calculating pooled RRs with 95% CIs by use of STATA 12.0 software (StataCorp, College Station, TX, USA). *P* < 0.05 was suggested to be statistically significant. The between-study heterogeneity was estimated by Cochran's *Q* and *I* ^2^ tests, and *P* < 0.05 and *I* ^2^ > 50% implicated obvious between-study heterogeneity [33, 34]. The random-effects model was used when the between-study heterogeneity was significant [35]; otherwise, the fixed-effects model was adopted [36]. Stratified analysis by study design (cohort studies, population-based case-control studies, and hospital-based case-control studies) was also performed. Sensitivity analysis by omission of each study was conducted for further analysis. Publication bias risk was evaluated by both Begg's funnel plots and Egger's test [37, 38].

## 3. Results

### 3.1. Identification and Characteristics of Studies Included into the Meta-Analysis

112 studies were retrieved after a comprehensive literature in databases of PubMed and Embase. However, 87 studies were excluded due to irrelevance, reviews, animal research, and case reports. 25 independent studies on the association between hormonal and reproductive factors and thyroid cancer risk were finally included into our study [8–32]. Among the 25 studies, 13 were cohort studies, 10 were population-based case-control studies, and the other 2 were hospital-based case-control studies. Characteristics of all included studies were summarized in Table 1.

### 3.2. Association between Hormonal Factors and Thyroid Cancer Risk

The common hormonal factors including oral contraceptive and hormone replacement therapy did not modify the risk of thyroid cancer (for oral contraceptive: RR = 0.94, 95% CI 0.85–1.04, *P* = 0.215; for hormone replacement therapy: RR = 1.04, 95% CI 0.91–1.19, *P* = 0.527) (Table 2). Sensitivity analysis by sequential omission of each study confirmed the findings (data not shown).

Stratified analysis by study design showed that no significant relationship was observed between hormonal factors and thyroid cancer risk in cohort studies and studies in population-based case-control design (Table 2). We failed to perform stratified analysis in hospital-based case-control studies because of insufficient published studies.

### 3.3. Association between Reproductive Factors and Thyroid Cancer Risk

**Figure 1:**
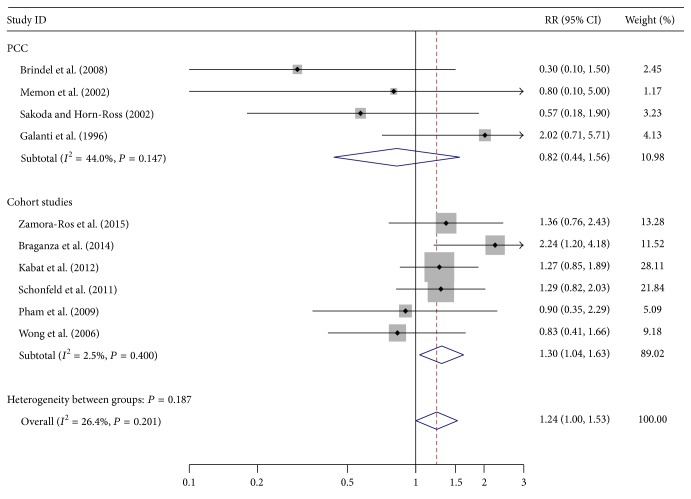
Forest plot for thyroid cancer risk related to age at menopause.

**Figure 2:**
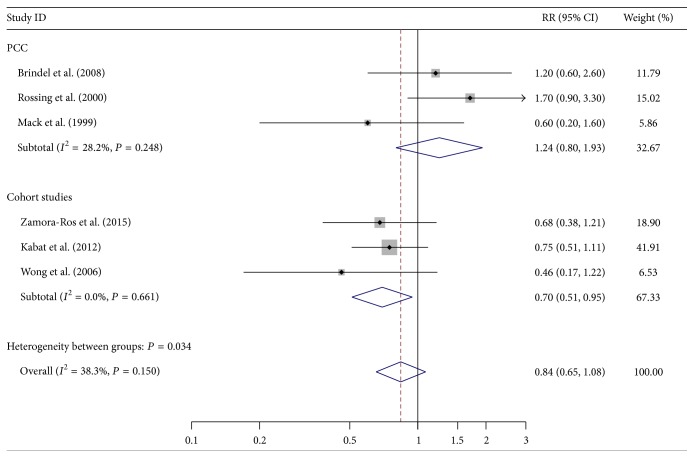
Forest plot for thyroid cancer risk related to duration of breastfeeding.

**Figure 3:**
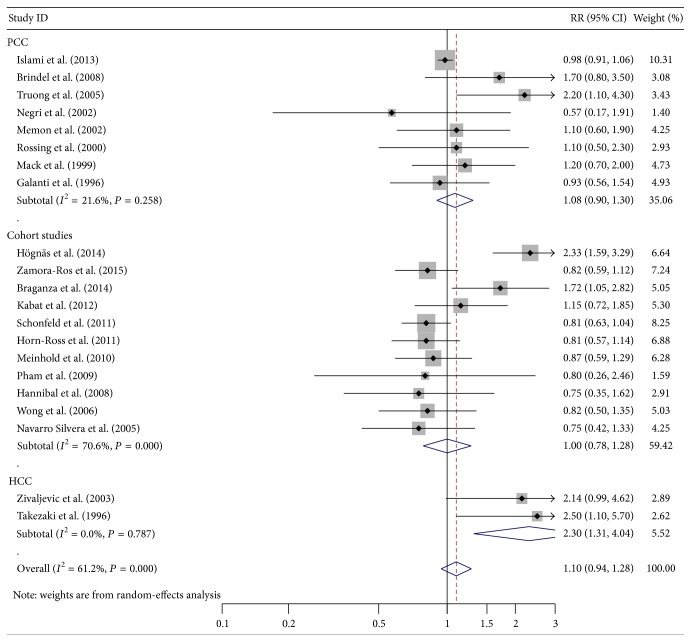
Forest plot for thyroid cancer risk related to parity.

The pooled RRs revealed that older age at menopause was associated with weakly increased risk of thyroid cancer in overall analysis (RR = 1.24, 95% CI 1.00–1.53, *P* = 0.049) (Table 2; Figure 1), whereas longer duration of breast feeding was related to moderately reduced risk of thyroid cancer in cohort studies (RR = 0.7, 95% CI 0.51–0.95, *P* = 0.021) (Table 2; Figure 2). Stratified analysis in hospital-based case-control studies showed that more parity could increase the risk of thyroid cancer (RR = 2.30, 95% CI 1.31–4.04, *P* = 0.004) (Table 2; Figure 3). No significant relationship was observed between thyroid cancer risk and other common reproductive factors (Table 2). Sensitivity analysis did not materially alter the pooled results (data not shown).

### 3.4. Heterogeneity Analysis and Publication Bias Risk

No significant between-study heterogeneity was found in most comparisons of overall and stratified analyses, except for the estimation of parity's effect on thyroid cancer risk (*I* ^2^ = 61.2%, *P* < 0.001). Stratified analysis by study design suggested that the main source of between-study heterogeneity resulted from studies in cohort design (*I* ^2^ = 70.6%, *P* < 0.001).

As suggested by Begg's funnel plots and Egger's test, there was no significant publication bias under all comparisons in the present meta-analysis (data not shown).

## 4. Discussion

Hormonal and reproductive factors have been implicated in the development of thyroid cancer, but the precise association and underlying molecular mechanisms have not yet been fully understood. A previous pooled analysis has investigated the association between female reproductive factors and thyroid cancer risk [39]. Unfortunately, only 17 epidemiological studies are included into the meta-analysis, which shows weak and equivocal association between some hormonal and menstrual cycle factors and thyroid cancer risk [39]. The present meta-analysis was based on 25 epidemiological studies on the association between hormonal and reproductive factors and thyroid cancer risk. No significant association was observed between thyroid cancer risk and common hormonal factors including oral contraceptive and hormone replacement therapy. Interestingly, older age at menopause might increase the risk of thyroid cancer, as suggested by the pooled RR in overall analysis (RR = 1.24, 95% CI 1.00–1.53, *P* = 0.049). Besides, longer duration of breast feeding was associated with moderately decreased risk of thyroid cancer, which had been suggested by the pooled analysis in cohort studies (RR = 0.7, 95% CI 0.51–0.95, *P* = 0.021). Moreover, the pooled result in hospital-based case-control studies revealed that parous women were more susceptible to thyroid cancer than nulliparous women (RR = 2.30, 95% CI 1.31–4.04, *P* = 0.004). Additionally, other reproductive factors including age at menarche, age at first birth, menopausal status, age at menopause, and breast feeding status did not modify the risk of thyroid cancer.

The risk of thyroid cancer in women increases at the time of puberty and declines after menopause [7, 40], supporting the hypothesis that menstrual cycle factors are involved in thyroid carcinogenesis. Elevated risk of thyroid cancer was related to the menopausal status when compared with premenopausal status [9]. Nonetheless, no significant association between the menopausal status, age at first birth, age at menarche, and age at menopause and thyroid cancer risk was observed [16, 18, 23, 27, 31]. It was worthwhile to note that menopausal females due to surgical factors were more susceptible to thyroid cancer compared with natural menopause ones [31], which might reflect enhanced medical surveillance of women who underwent surgical interventions for gynecological diseases symptoms. Similarly, we failed to identify any appreciable relationship of menstrual factors with thyroid cancer susceptibility. The discrepancies and underlying mechanisms need to be further elucidated by more relevant studies in the future.

It has been well established that estrogen receptors are found in thyroid cancer tissue and confer effects on different molecular signaling pathways involved in the growth and function of thyroid [5, 41, 42]. Estrogens and estrogen receptors signals exert promoting effect on the growth of thyroid gland by enhancing levels of TSH [7]. A number of studies have suggested some hormonal related factors, for instance, oral contraceptive and hormone replacement therapy, which played different roles in the thyroid cancer risk [8, 23, 27, 31]. Oral contraceptive seemed to play a protective role against thyroid cancer while an increased risk of thyroid cancer was found with the use of hormone replacement therapy demonstrated by Zamora-Ros et al. [31]. Similar findings were elucidated in a Caucasian cohort study [27]. Conversely, no modifying effects of oral contraceptive and hormone replacement therapy on the development of thyroid cancer were in another independent cohort study [8, 23]. The contradictory findings may be attributed to different study design, use of contraceptive and hormone replacement therapy, ethnicity, and adjusted or matching criteria in individual epidemiological studies. Our study showed no appreciable roles of hormonal factors in thyroid carcinogenesis, as suggested by both overall analysis and stratified analysis according to study design. More future studies are warranted to further estimate the association between hormonal-related factors and thyroid cancer risk.

The effect of breastfeeding on thyroid cancer risk is still not clear. Kabat and colleagues demonstrated that duration of breastfeeding did not alter the susceptibility to thyroid cancer [16]. However, Mack et al. provided the evidence that longer duration of breastfeeding was negatively associated with the risk of thyroid cancer risk, suggesting a protective role of breastfeeding in thyroid carcinogenesis [17]. Similarly, the pooled RRs in all cohort studies implicated that longer duration of breast feeding was associated with moderately reduced risk of thyroid cancer. Nevertheless, there was no significant relationship between breastfeeding status and thyroid carcinogenesis in pooled analyses of total studies and population-based case-control ones. Thus, the moderate association might be a chance resulting from potential bias in the present meta-analysis. To better understand the role of breastfeeding status in the thyroid cancer development, more relevant studies with high quality are warranted.

The status of parity conferred diverse effects on thyroid cancer risk in different populations. A recent study by Braganza et al. showed that parous women were at an elevated risk of thyroid cancer, as suggested by a recent epidemiological study [8]. Interestingly, for any given level of parity, there was about twofold increased risk of thyroid cancer among women with the age at last pregnancy larger than 30 years [19]. Unlike the findings mentioned above, no appreciable association was suggested between parity and the susceptibility to thyroid cancer among a Caucasian population [27]. In the current meta-analysis, significantly positive association was only demonstrated in the pooled analysis of two hospital-based case-control studies. Although age seemed to influence roles of reproductive factors in thyroid carcinogenesis, we failed to find appreciable association for age at first birth, age at menarche, and age at menopause. In addition, the pooled results, particularly in hospital-based case-control studies, must be interpreted with caution due to limited sample size and insufficient statistical power in current research.

Age is a main confounding factor for the association between hormonal and reproductive factors and thyroid cancer risk [19]. We failed to perform stratified analysis by age or other confounding factors, such as dosage and usage of hormonal drugs, the reason of menopause, number of live births, outcome of first pregnancy, and history of miscarriage, because of unavailable information about these items in single studies. Consequently, the association between hormonal and reproductive factors and thyroid cancer risk should be further investigated in view of above-mentioned confounding factors.

## 5. Conclusions

The current meta-analysis suggests that older age at menopause and parity are associated with increased risk of thyroid cancer, while longer duration of breast feeding plays a protective role against this cancer. In addition, the precise association needs further investigation by more epidemiological studies with sufficient statistical power in the future.

## Figures and Tables

**Table 1:** Characteristics of all epidemiological studies.

First author	Year	Origin	Time	Study design^§^	Hormonal or reproductive factors	Adjusted or matching criteria
Högnäs [13]	2014	Finland	1974–2010	Cohort study	Parity	Sex

Zamora-Ros [31]	2015	Europe	1992–2009	Cohort study	Oral contraceptive, hormone replacement therapy, age at menarche, parity, age at first birth, menopausal status, age at menopause, breastfeeding status, and duration of breastfeeding	Sex, center, and age at recruitment

Braganza [8]	2014	USA	1993–2009	Cohort study	Oral contraceptive, hormone replacement therapy, age at menarche, parity, age at first birth, and age at menopause	Sex, education, race, marital status, family history of thyroid cancer, baseline body mass index, and smoking

Islami [15]	2013	Iran	2003–2007	PCC	Parity	Age, sex, and neighborhood of residence

Kabat [16]	2012	USA	1993–2011	Cohort study	Oral contraceptive, hormone replacement therapy, age at menarche, parity, age at first birth, age at menopause, and duration of breastfeeding	Sex, age, education, height, history of goiter/nodules, pack-years, and alcohol intake

Schonfeld [27]	2011	USA	1995–2006	Cohort study	Oral contraceptive, hormone replacement therapy, age at menarche, parity, age at first birth, and age at menopause	Sex, smoking status, baseline body mass index, race, alcohol consumption, and education

Horn-Ross [14]	2011	USA	1995–2008	Cohort study	Oral contraceptive, hormone replacement therapy, age at menarche, parity, age at first birth, and menopausal status	Sex, ethnicity, family history of thyroid cancer, time since last pregnancy, smoking, alcohol consumption, physical inactivity, height, adolescent cycle length, time to regular menstruation, and age at menarche

Meinhold [18]	2010	USA	1983–2006	Cohort study	Oral contraceptive, hormone replacement therapy, age at menarche, parity, and menopausal status	Sex, birth year, smoking status, body mass index, number of personal radiographs to the head or neck, and cumulative occupational radiation dose

Pham [23]	2009	Japan	1988–1997	Cohort study	Hormone replacement therapy, age at menarche, parity, age at first birth, and age at menopause	Sex, age at baseline, body mass index, tobacco smoking status, education level, history of diabetes, and study area

Dorjgochoo [10]	2009	China	1996–2006	Cohort study	Oral contraceptive	Sex, education, age at menarche, number of live births, cumulative breast feeding months, body mass index, exercised regularly in past 5 years, smoking, menopausal status, first-degree family history of cancer, and other contraceptive methods

Hannibal [12]	2008	Denmark	1963–2000	Cohort study	Parity and age at first birth	Sex, parity, age at cohort entry and calendar year of cohort entry, and age at first live birth

Brindel [9]	2008	France	1981–2004	PCC	Age at menarche, parity, age at first birth, menopausal status, age at menopause, breastfeeding status, and duration of breastfeeding	Sex, educational level, height, body mass index, and interviewer

Wong [30]	2006	China	1989–1998	Cohort study	Oral contraceptive, age at menarche, parity, age at first birth, age at menopause, breastfeeding status, and duration of breastfeeding	Sex, age, number of live births, and age at first live delivery

Truong [29]	2005	New Caledonia	1993–1999	PCC	Oral contraceptive, hormone replacement therapy, age at menarche, and parity	Sex, frequency, age, and residential area

Neale [21]	2005	Sweden	1961–1996	Cohort study	Age at first birth	Sex, date of birth of the mother, parity, and age at first birth

Navarro Silvera [20]	2005	Canada	1980–2000	Cohort study	Oral contraceptive, hormone replacement therapy, age at menarche, parity, age at first birth, and menopausal status	Sex, age at first live birth, study center and randomization group, parity, and HRT use

Zivaljevic [32]	2003	Serbia	1996–2000	HCC	Parity	Sex, age, place of residence, and time of hospitalization

Negri [22]	2002	Mixed	1974–1992	PCC	Age at menarche, parity, age at first birth, and menopausal status	Sex, age, ethnicity, and study center

Memon [19]	2002	Middle East	1981–1999	PCC	Oral contraceptive, hormone replacement therapy, age at menarche, parity, age at first birth, and age at menopause	Sex, year of birth, nationality, and district of residence.

Sakoda [26]	2002	USA	1992–1998	PCC	Age at menarche, menopausal status, and age at menopause	Sex, frequency, age, ethnicity, education level, history of goiter or nodules, history of radiation to the head or neck, and family history of proliferative thyroid disease

Rossing [24]	2000	USA	1988–1994	PCC	Age at menarche, parity, age at first birth, and duration of breastfeeding	Sex, age, county of residence, race, and relative weight at age 10

Mack [17]	1999	USA	1980–1983	PCC	Age at menarche, parity, age at first birth, menopausal status, breastfeeding status, and duration of breastfeeding	Sex, age, prior thyroid disease, and number of births

Takezaki [28]	1996	Japan	1988–1993	HCC	Age at menarche, parity, and age at first birth	Sex, age, and year of visit

Rossing [25]	1998	USA	1988–1994	PCC	Oral contraceptive and hormone replacement therapy	Sex, age, county of residence, race, and relative weight at age 10

Galanti [11]	1996	Sweden and Norway	1985–1993	PCC	Oral contraceptive, hormone replacement therapy, parity, age at first birth, and age at menopause	Sex, frequency, year and month of birth, type of menopause, and parity

^§^PCC: population-based case-control studies; HCC: hospital-based case-control studies.

**Table 2:** Summary results for the association between hormonal and reproductive factors and thyroid cancer risk.

Comparisons	^a^RR	^b^95% CI	^c^ *P* value	Tests for heterogeneity	
				*I* ^2^ (%)	^d^ *P*
All					
Oral contraceptive	0.94	0.85–1.04	0.215	36.0	0.095
Hormone replacement therapy	1.04	0.91–1.19	0.527	0	0.520
Age at menarche	1.08	0.97–1.19	0.142	0	0.486
Parity	1.10	0.94–1.28	0.234	61.2	<0.001
Age at first birth	1.07	0.95–2.20	0.255	14.8	0.281
Menopausal status	0.96	0.79–1.18	0.727	4.9	0.394
Age at menopause	1.24	1.00–1.53	0.049	26.4	0.201
Duration of breast feeding	0.84	0.65–1.08	0.178	38.3	0.150
Breast feeding	0.84	0.69–1.02	0.080	4.9	0.368
Cohort studies					
Oral contraceptive	0.96	0.86–1.07	0.448	39.5	0.104
Hormone replacement therapy	1.05	0.91–1.20	0.513	0	0.439
Age at menarche	1.01	0.90–1.14	0.808	16.1	0.299
Parity	1.00	0.78–1.28	0.993	70.6	<0.001
Age at first birth	1.04	0.92–1.18	0.530	0	0.571
Menopausal status	0.90	0.71–1.15	0.418	26.6	0.252
Age at menopause	1.3	1.04–1.63	0.022	2.5	0.400
Duration of breast feeding	0.70	0.51–0.95	0.021	0	0.661
Breast feeding	0.82	0.66–1.02	0.078	0	0.442
PCC					
Oral contraceptive	0.89	0.73–1.08	0.240	41.0	0.166
Hormone replacement therapy	1.01	0.68–1.50	0.962	6.0	0.363
Age at menarche	1.28	1.04–1.57	0.960	0	0.937
Parity	1.00	0.93–1.07	0.960	21.6	0.258
Age at first birth	1.35	0.97–1.88	0.072	42.3	0.123
Menopausal status	1.10	0.78–1.57	0.577	0	0.481
Age at menopause	0.82	0.43–1.56	0.554	44.0	0.147
Duration of breast feeding	1.24	0.80–1.93	0.332	28.2	0.248
Breast feeding	0.92	0.60–1.41	0.699	57.6	0.125
HCC					
Parity	2.30	1.31–4.04	0.004	0	0.787

^a^RR: relative risk; ^b^95% CI: 95% confidence interval; ^c^ *P*: the value of *P* for pooled analysis; ^d^ *P*: the value of *P* for heterogeneity analysis.
